# The association between cannabis use and cognitive performance in older adults: findings from the UK biobank

**DOI:** 10.1002/alz70860_105630

**Published:** 2025-12-23

**Authors:** Sharon Sznitman, Shiraz Vered, Galit Weinstein

**Affiliations:** ^1^ University of Haifa, Haifa, Israel; ^2^ School of Public Health, University of Haifa, Haifa, Mount Carmel, Israel

## Abstract

**Background:**

Cannabis may affect the aging brain differently compared to younger populations, yet the long‐term cognitive effects of cannabis use in advanced age are unclear. Our study investigated the cross sectional and longitudinal relationships between past and present cannabis consumption and cognitive function in dementia‐free adults aged ≥60 years in the UK biobank study.

**Method:**

This cross‐sectional and longitudinal study included participants of the UK biobank who completed online self‐assessment questionnaires, including inquiries about cannabis consumption. Performance on attention, executive function, processing speed, visual memory and working memory were extracted from the UK Biobank dataset. Multivariable linear regression models were employed to assess the relationship between cannabis use patterns and cognitive performance, while adjusting for potential confounders, including sociodemographic and life‐style factors. Longitudinal associations were assessed by including the second follow‐up cognitive scores as dependent variables, entering first cognitive measures and inter‐assessment duration as additional covariates.

**Result:**

The sample included 67,713 participants (mean age 67.2±4.4y, 46.1% men), 17% reported past cannabis use and of them, 4% reported current use. Former vs. never cannabis users performed better on cognitive tests (β=0.069±0.005; *p* <0.001, β=0.046±0.004; *p* <0.001, β=0.345±0.051; *p* <0.001, β=0.060±0.014; *p* <0.001, β=0.186±0.016; *p* <0.001 for attention, executive function, processing speed, visual memory and working memory, respectively). Current vs. never users performed better on working memory (β=0.191±0.069; *p* = 0.006). Among former users, frequent cannabis use was associated with better attention (β=0.026±0.009; *p* = 0.003) and starting before age 17y was associated with better attention and visual memory (β=0.092±0.014; *p* <0.001 and β=0.115±0.045; *p* = 0.011, respectively) but with worse working memory (β=‐0.148±0.049; *p* = 0.003). Former users reporting cannabis use for more >5 years had slower processing speed (β=‐0.0256±0.109; *p* = 0.019) than those reporting shorter duration. Among former users, longer duration was associated with slower processing speed over time (β=‐0.248±0.116; *p* = 0.033).

**Conclusion:**

Our findings suggest that former and current cannabis use may not have widespread negative effects on cognition in older individuals. The observed positive associations between cannabis use and cognition in older adults may be due to cannabis compounds with neuroprotective properties. Additionally, cannabis use in older adults may indicate social engagement and a more active lifestyle, known to positively affect cognitive health.

